# Positive emotions in fairy-tale reading: a text-mining study of nostalgia among adult purchasers from 1,428 online reviews

**DOI:** 10.3389/fpsyg.2026.1797031

**Published:** 2026-04-10

**Authors:** Caizhen Liu, Tangying Feng, Jing Wang

**Affiliations:** 1 School of Humanities and International Communication, Ningbo, Zhejiang, China; 2 College of Child Development and Education, Zhejiang Normal University, Hangzhou, Zhejiang, China

**Keywords:** Chinese culture, fairy tales, nostalgia, online reviews, psychological ownership, reading motivation, text mining

## Abstract

Despite the popularity of Chinese classic fairy tales, research on the psychological associations of reading such stories remains limited, especially regarding the discursive expressions and functions of nostalgia. This study explores the association between nostalgia and the reported experience of reading Chinese classic fairy tales among adult purchasers. Taking the classic “Chinese Fairy Tales” (12 volumes) on Dangdang.com as a case, we collected 1,428 purchaser reviews through crawler technology. Text-mining and sentiment analysis were employed to analyze purchasers’ reported reading experiences and identify psychological factors specifically related to fairy-tale reading. Findings reveal that nostalgia emerges as a prominent discursive theme in adult purchasers’ reviews when discussing their selection of fairy tales for children. The theory of readers’ nostalgia and psychological ownership—encompassing five aspects: occupying a position, self-efficacy, self-identity, responsibility, and territoriality—is clearly reflected in the analyzed discourse. This study highlights the role of nostalgia in adult purchasers’ book-selection motivations and offers an exploratory mapping of psychological ownership themes in the context of nostalgic reading.

## Introduction

Fairy tales serve as vital psychological tools, helping children navigate inner conflicts, anxieties, and desires. While Western scholarship has long debated the effects of fairy tales—with early critics like Felix Adler and Maria Montessori warning of “mental confusion” while proponents like Bruno Bettelheim emphasized their “therapeutic value”—few studies have quantitatively examined the discursive representation of traditional Chinese fairy tales among contemporary readers. With approximately 298 million children in China as of the 2020 Census, the role of fairy tales in cultural transmission and emotional development is of significant social importance. Most existing research has focused on Western narratives, leaving a gap in our understanding of how Chinese readers resonate with their own cultural folklore. Furthermore, the role of adult purchasers—who act as “gatekeepers” to children’s reading—remains under-investigated. The specific ways in which adult nostalgia is associated with purchasing decisions and the perceived facilitation of reading have not been explored through large-scale data analysis. To address these gaps, the present study utilizes the “Chinese Fairy Tales” collection (a 12-volume set compiled over 10 years and covering 362 original stories) as a case study. By analyzing thousands of user-generated reviews from Dangdang, China’s largest online bookstore, we seek to uncover the self-reported psychological experiences of buyers. This study addresses the research problem of how adult nostalgia and psychological ownership are discursively articulated in the self-reported reviews of traditional fairy tales. Our specific objectives are: (1) to quantify the emotional valence of adult readers toward classic Chinese fairy tales; (2) to identify the specific dimensions of psychological ownership reflected in reader discourse; and (3) to evaluate how nostalgic narratives in e-commerce reviews conceptually align with themes of cultural and media psychology. This research provides the first data-driven exploratory mapping of psychological ownership vocabulary in nostalgic reading discourse within the Chinese cultural context.

### Literature review

Research into the psychological effects of fairy tales on children has generated lasting interest in academia. Since the 1920s, there has been a heated debate regarding whether they have positive or negative effects. Some psychiatrists, such as Felix Adler, recommended their elimination due to “harmful, superstitious, and immoral elements” (Eddy, 2006). Montessori likewise crusaded against them, declaring that they “plunge the child into the supernatural” and create a “dread of reality” (Eddy, 2006). Conversely, a professor at Clark University suggested that fairy tales provide “compensation for being little and helpless,” allowing children to realize their own desires (Child Study Association of America, 1927).

Other scholars contend that fairy tales, as a favorite literary style, align with children’s psychological development. They have constructive value, fulfilling wishes much like the structure of dreams (Lorand, 1935). However, Benjamin Gruenberg warned that fairy tales might represent “abnormal gratification,” prolonging the “wishing stage” and preventing children from exerting the “real effort” necessary for life goals (Child Study Association of America, 1927). Notably, a group of female scholars challenged these views, arguing that fairy tales, as imaginative literature, reflect the “larger reality of human existence” (Jenkins, 1996). They suggested that reading should focus on excellent qualities like courage and loyalty rather than individual details, linking fairy tale writers to poets who offer “intellectual honesty and spiritual clarity” (Eddy, 2006; Sawyer, 1962).

In the 1970s, Bettelheim (1977) argued that the messages of fairy tales are complex and of great value, helping children cope with inevitable fears such as abandonment. Fairy tales also serve as a means of cultural transmission, reflecting collective folk wisdom and moral teachings (Zipes, 2006). While critics argue that evidence for their harm is lacking (Katz Lilian, 1991), the therapeutic aspect is evident in narratives that emphasize emotional support and the presence of loving figures (Karbowniczek and Kucharska, 2020).

In the 1990s, psychological applications became more widespread, from easing pain in dental procedures (Ishikawa et al., 1990) to the creation of the Fairy Tale Test (FTT) (Coulacoglou, 2000). During the COVID-19 pandemic, new fairy tales emerged to help children process anxieties by portraying the virus as a character, providing a framework for understanding their environment (Karbowniczek and Kucharska, 2020). Today, fairy tales serve as integral tools for navigating emotions and moral dilemmas (Tsitsani et al., 2012; Girsang et al., 2023). Evidence suggests they stimulate imagination and autonomy more effectively than everyday experiences (Rokotnitz, 2018), with children showing a consistent preference for traditional narratives (Karbowniczek and Kucharska, 2020).

Despite this rich history, academic discourse remains largely Western-centric, with little attention given to the influence of Chinese fairy tales on Chinese readers. Specifically, few studies have investigated how these tales impact children’s psychology through quantitative research. Given that China’s 2020 census identified 298 million children (National Bureau of Statistics of China, 2021), understanding how they absorb these messages is crucial. This study employs content analysis—a systematic method proven effective in large-scale textual research (Liu et al., 2022)—to explore experiential data from the Dangdang platform. We focus on the classic *Chinese Fairy Tales*, a highly awarded 12-volume set compiled over a decade, which has become a “family heirloom” since its mainland introduction in 2018, providing a unique case study for analyzing long-term reader engagement and psychological resonance.

Research and design using a web crawler, readers’ comments on China Fairy Tales on the Dangdang website from June 17, 2018, to April 25, 2024, were statistically analysed, the emotional reading experience of Chinese fairy tales was scored, and the factors affecting Chinese readers’ reading were subsequently coded and analysed. To identify the most authentic reading experience and psychological factors of Chinese readers, they read fairy tales.

Data collection to obtain the review data of designated books on Dangdang, we designed and implemented a Python-based crawler program. The program automatically captures the review information of books in a multistep way and stores it as an Excel file. The specific extraction target was Hansheng Chinese Fairy Tales (ISBN: 978-7-5554-3105-3, Product ID [PID]: 29464061). The following is the specific implementation process of the crawler:

Import the required libraries: First, we import the necessary Python libraries: requests are used to send HTTP requests to Dangdang servers to obtain web page data. Beautiful soup is used to parse the HTML of a web page and extract the required information. Pandas is used to store and process comment data and eventually export it as an Excel file.

To ensure that crawlers can successfully access Dangdang and avoid being identified by the website as machine requests, we set HTTP headers and cookies in the request. This information simulates the request behavior of normal users to ensure that the crawler can successfully access the target web page.

We specify the URL of the Dangdang book review page and set the request parameters. The original targeted review endpoint followed this structural pattern http://product.m.dangdang.com/review.php?action=get_review_info&pid=29464061&shop_id=0&is_book=1&main_pid=0&product_medium=0&pod_pid=&category_id=50006&category_path=01.41.26.01.00.00. The crawler crawled the review page by page through paging and obtained all the relevant review data. This process iterates 96 times, traversing all the comment pages.

GET comment information: On each page, the crawler sends a GET request and obtains the response data. The response data are in HTML format, and we use beautiful soup to parse the HTML content. During this process, we specifically extracted data across exactly four dimensions: Username: The username of the commenter, obtained by parsing the <p class=‘name’> tag in HTML. Comment score: The rating given by the reviewer parses the rating data in the <div class=‘stars’> tag in HTML. Comment content: The specific feedback content of the reviewer extracts the text in the <p class=‘review_text’> tag. Comment time: When the comment was published, the date information in the <span class=‘date’> tag was parsed.

We acknowledge several methodological limitations regarding the computational reproducibility of this scraping process. First, due to equipment and structural transitions over the extended collection period (June 2018–June 2024), specific operational parameters—including exact HTTP request intervals (estimated at a 2–5 s delay) and the full original codebase—were not systematically preserved. Second, it is important to clarify that our dataset does not include a “verified purchase” metric. This is not due to an extraction omission, but rather because Dangdang’s front-end review architecture inherently lacked a designated “verified purchase” feature or badge during the target epoch.

Despite these technical constraints, the core textual and demographic fields were successfully secured in an offline, de-identified dataset. Each time a comment is extracted, the crawler stores it in a Pandas DataFrame. This dataframe contains four fields: username, comment score, comment content, and comment time. By adding these data to the DataFrame one by one, we are able to organize and manage the captured comment data effectively.

After all the comment data have been successfully crawled, we use the pandas to_Excel() method to save the data in an Excel file. The file is named Review.xlsx and does not contain the row index. The finalized dataset comprises 1,428 extracted records encoded in UTF-8. To promote transparency and align with FAIR (Findability, Accessibility, Interoperability, and Reusability) data practices, this offline dataset will be archived and made available to researchers upon request.

Finally, the program prints “Data saved to review.xlsx file”, which shows that the crawler task has been successfully completed.

From June 17, 2018, to June 14, 2024, the crawler collected 1,428 comments. These comments were coded, and nine aspects were obtained, as shown in Table 1:

**Table 1:** Book purchase and reading experience feedback statistics.

Category	Frequency	Specific content
Packing	134	Packing issues of books
Quality of content	113	The quality of book content
Traditional culture	71	Traditional cultural elements in books
Price	66	The price or discounts when purchasing books
Express service	65	The quality of express services
Motivation to buy	60	Reasons for buying books
Book weight	49	The weight or portability of books
Illustration quality	30	The quality of book illustrations
Parent–child reading	13	The experience of parent–child reading

The table shows that consumers pay the most attention to the book’s packaging, content quality and traditional cultural elements when evaluating Chinese Fairy Tales while also taking into account factors such as price, express delivery service and purchase motivation. This information provides valuable feedback for publishers and sales platforms to help them optimize products and improve service quality.

The reader’s psychological experience with fairy tales is included in the 1,428 comments, so the text can be analysed via the free software KHCoder. This approach has reproducibility of analysis results through automatic operation and avoids the incorporation of researcher prejudices (Hirahara, 2021).

**Figure 1:**
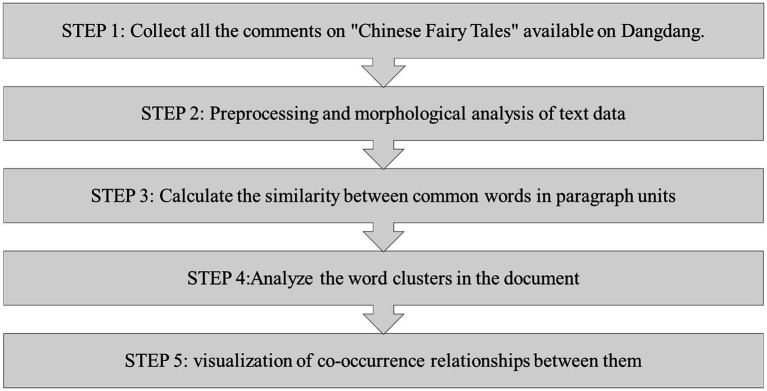
Flowchart showing the steps of this research.

Figure 1 Specific research steps are as follows: Step 1: Collect and digitize all the comments published in Dangdang’s China Fairy Tales from 2018 to 2024. The text analysis is aimed at Chinese, as the article uses Chinese as the original language. However, for writing this article, the morphemes in the text data were translated into English. Step 2: Preprocess and analyse the word frequency table. Step 3: 116 commonly used words are listed, and the co-occurrence relationships between them are visualized in the network diagram. The graph consists of the first 70 relationships that use the Jaccard index to calculate the similarity between common words in paragraph units. Step 4: Perform an analysis of the word clusters in the document. The 10 word clusters of the document content explain the participants’ experiences and perceptions of fairy tale reading. Step 5: Word co-occurrence network analysis. The co-occurrence network construction technique offers a visual method to display key individuals, organizations, and concepts present in the text, as well as to uncover the potential links among these prominent terms. This technology operates on the principle of identifying the most commonly used words within a text through word frequency analysis. It then connects words that frequently cooccur with straight lines to create a network graph. In this graph, words that appear more frequently are represented by larger circles (Mao, 2019). This graph intuitively shows the high-frequency words in the text and the relationships among them. These high-frequency words often show hot events in the text, and the relationships between the words reflect some important information about these events.

## Results

**Figure 2:**
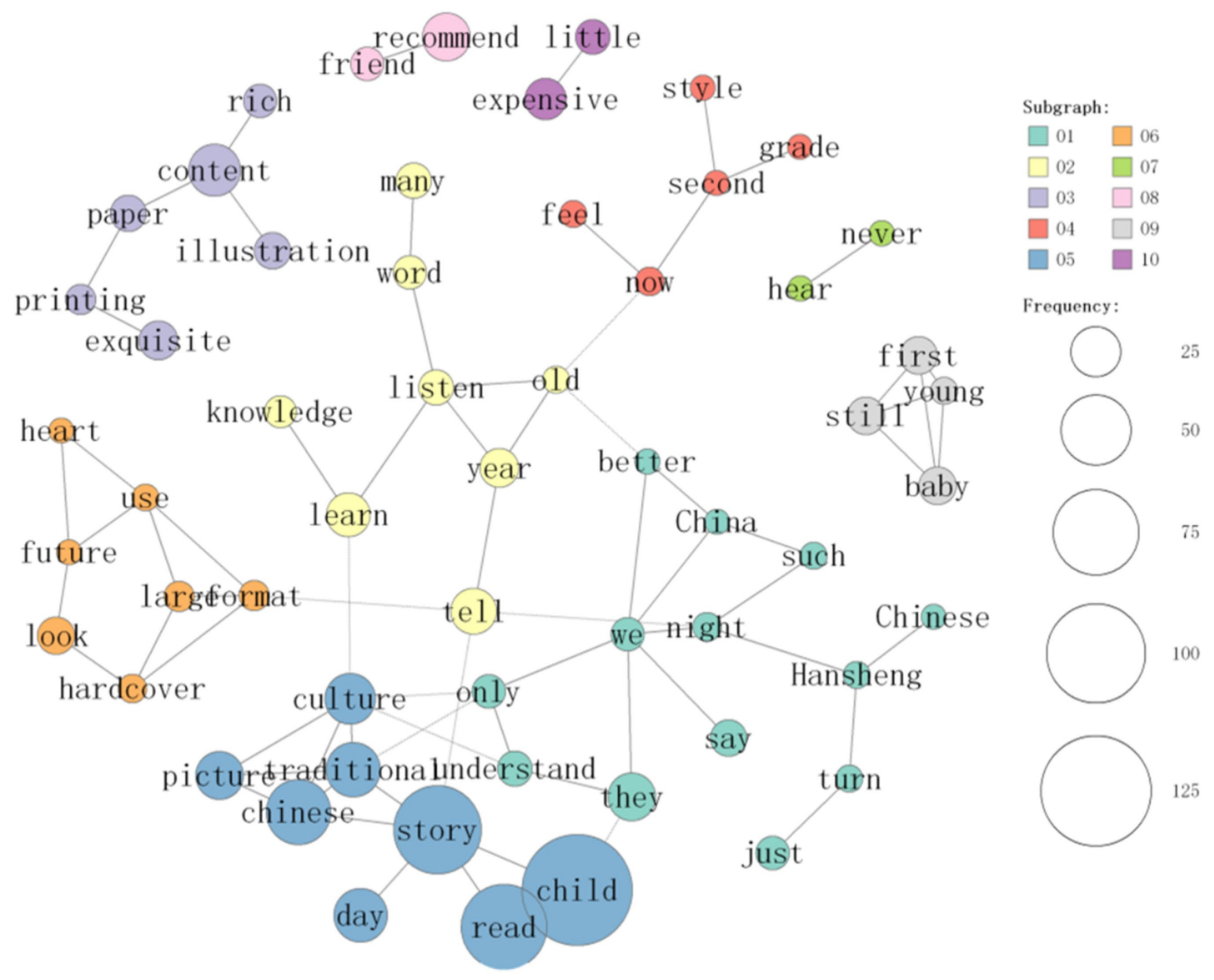
Display co-occurrence diagram of one of the word clusters.

**Figure 3:**
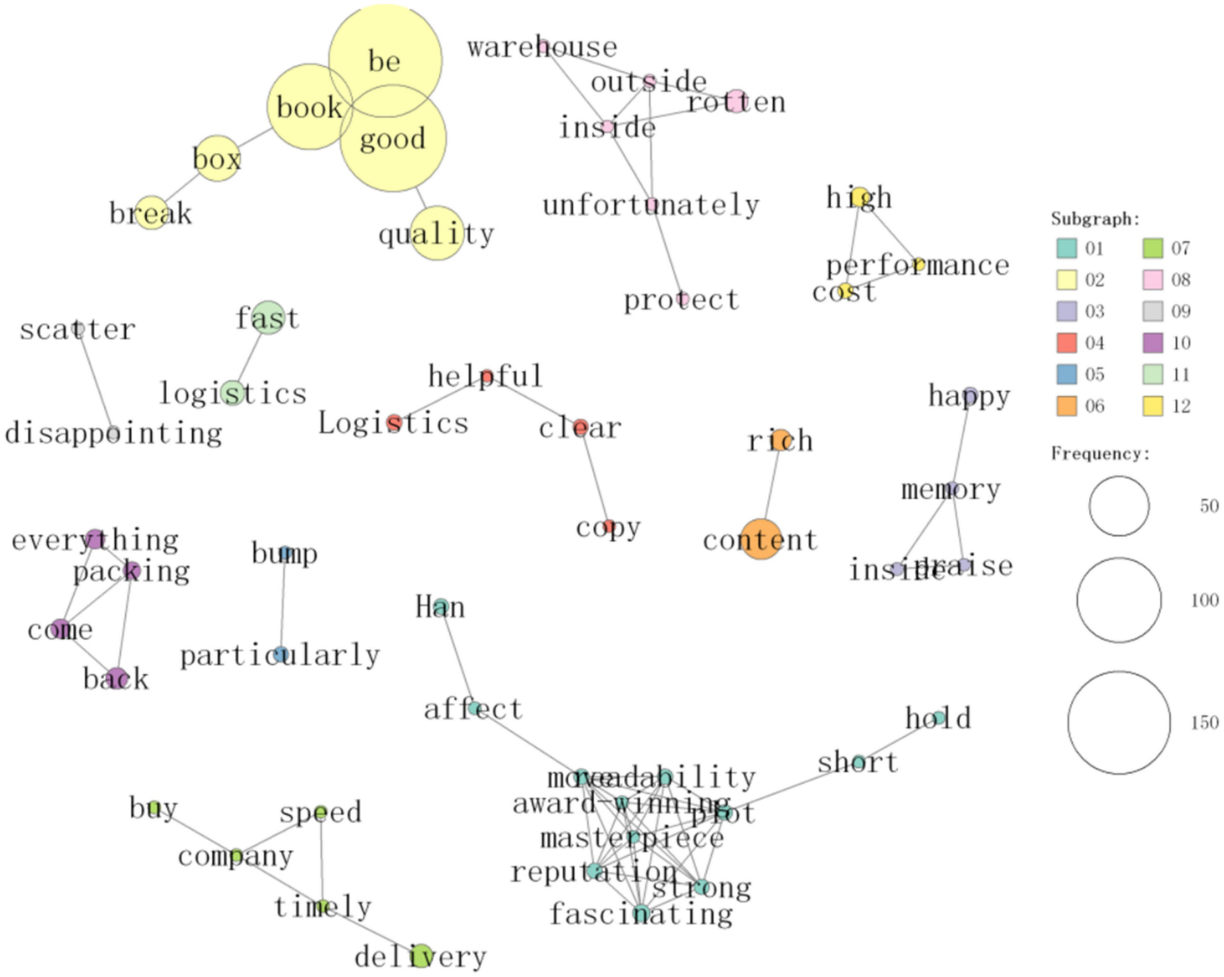
Display cooccurrence diagram of one of the word clusters.

On the basis of the analysis of 1,428 comments, the co-occurrence network of frequent words related to reading experience in the comments of “Chinese Fairy Tales” was constructed via the built-in “co-occurrence network” function of KH Coder, as shown in Figures 2, 3. To ensure methodological transparency and reproducibility, the specific parameters for this network generation and subsequent clustering were configured as follows: the unit of analysis for calculating co-occurrence was set to the paragraph level. Furthermore, the vocabulary was filtered to include meaningful parts of speech (e.g., nouns, proper nouns, adjectives, adverbs, and verbs), with a minimum word frequency threshold (PF) set to 5 to eliminate background noise.

Figures 2, 3 illustrates this co-occurrence network that describes correlations between frequently used words. The node size is proportional to the word frequency; the edge thickness is proportional to the similarity calculated via the Jaccard index. To maintain visual clarity, the network was constrained to display only the top 70 strongest relationships (edges). By observing the co-occurrence network in Figures 2, 3, the high-frequency words were statistically clustered into 10 distinct sub-groups using KH Coder’s clustering analysis algorithm based on Jaccard distance (visually distinguished by different node colors/boundaries). These high-frequency word groups reveal the multifaceted reading experience of Chinese Fairy Tales. Positive comments such as “good” and “recommend” appear prominently in the high-frequency words, indicating that the reading experience of fairy tales is full of affirmation.

To verify the adult readers’ expressed emotional experience of reading the book, we calculated these comments via the emotional score method. First, we stored the comment data in CSV format, and each CSV file corresponds to a specific evaluation dimension (e.g., parent–child, traditional culture, or experience). The files were preprocessed to ensure that they had a clear structure and that each comment had been cleaned for subsequent sentiment analysis. Unnecessary columns were deleted; we kept only the comment column to ensure that the data were clear and concise.

Then, SnowNLP was used as a tool for sentiment analysis. The sentiment score ranges from 0 to 1, where a value close to 0 indicates that the comment sentiment is negative, and a value close to 1 indicates more positive emotion. ~ ~ whereas a value closer to 1 indicates more positive emotion. ~~Positive emotion comments contain positive words such as “satisfaction,” “well,” and “like,”~~and “satisfaction,”~~ whereas a value closer to 0 indicates more negative emotions. Negative comments contain negative words such as “dissatisfaction,” “poor,” “disappointment,” “bad” and “problem.”

To address the inherent challenges of automated sentiment analysis in e-commerce contexts—such as identifying sarcasm, mixed emotions, or complex negations—we conducted a rigorous manual validation before calculating the final dimensions. A random subset of exactly 100 reviews was extracted and blindly annotated by human coders as positive (1) or negative (0). Comparing these ground-truth labels against the SnowNLP binary outputs (using a strict 0.5 threshold) yielded an accuracy of 91.0, indicating that our sentiment classification approach is sufficient to support the analytical objectives of this exploratory study. Qualitative error inspection revealed that while the algorithm occasionally misclassified complex mixed sentiments (e.g., reviews praising the book content but heavily criticizing the postal delivery service) and sentences with double negations, its performance was exceptionally reliable for identifying general emotional polarity. Therefore, the generated scores accurately map macroscopic sentiment trends across the broader dataset.

After the sentiment of all the comments in each CSV file was scored, the average sentiment score of nine aspects, including packaging, content quality, traditional culture and price, was calculated. The average sentiment score represents the sentiment tendency of the whole dataset.

**Figure 4:**
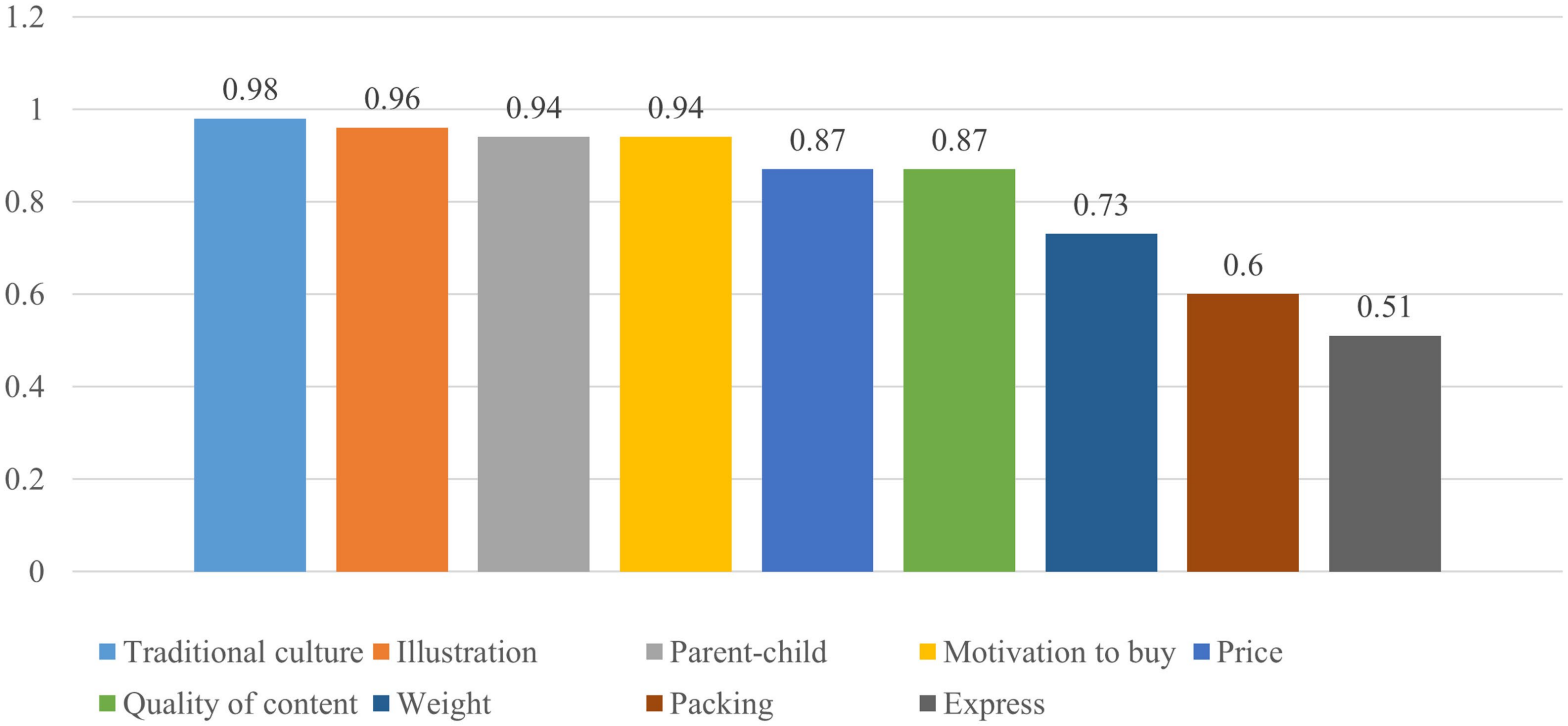
Emotional rating scales for different aspects.

The results obtained via calculation are shown in Figure 4.

The results of calculating the average value indicate that Dangdang readers’ emotional score for “Chinese Fairy Tales” is far greater than 0.5, close to 1, and its emotional evaluation is positive. Through this method, we can understand the emotional distribution and tendency of Dangdang book reviews and analyse the user feedback of different book types or topics to better understand adult purchasers’ self-reported emotional response to the book content.

**Figure 5:**
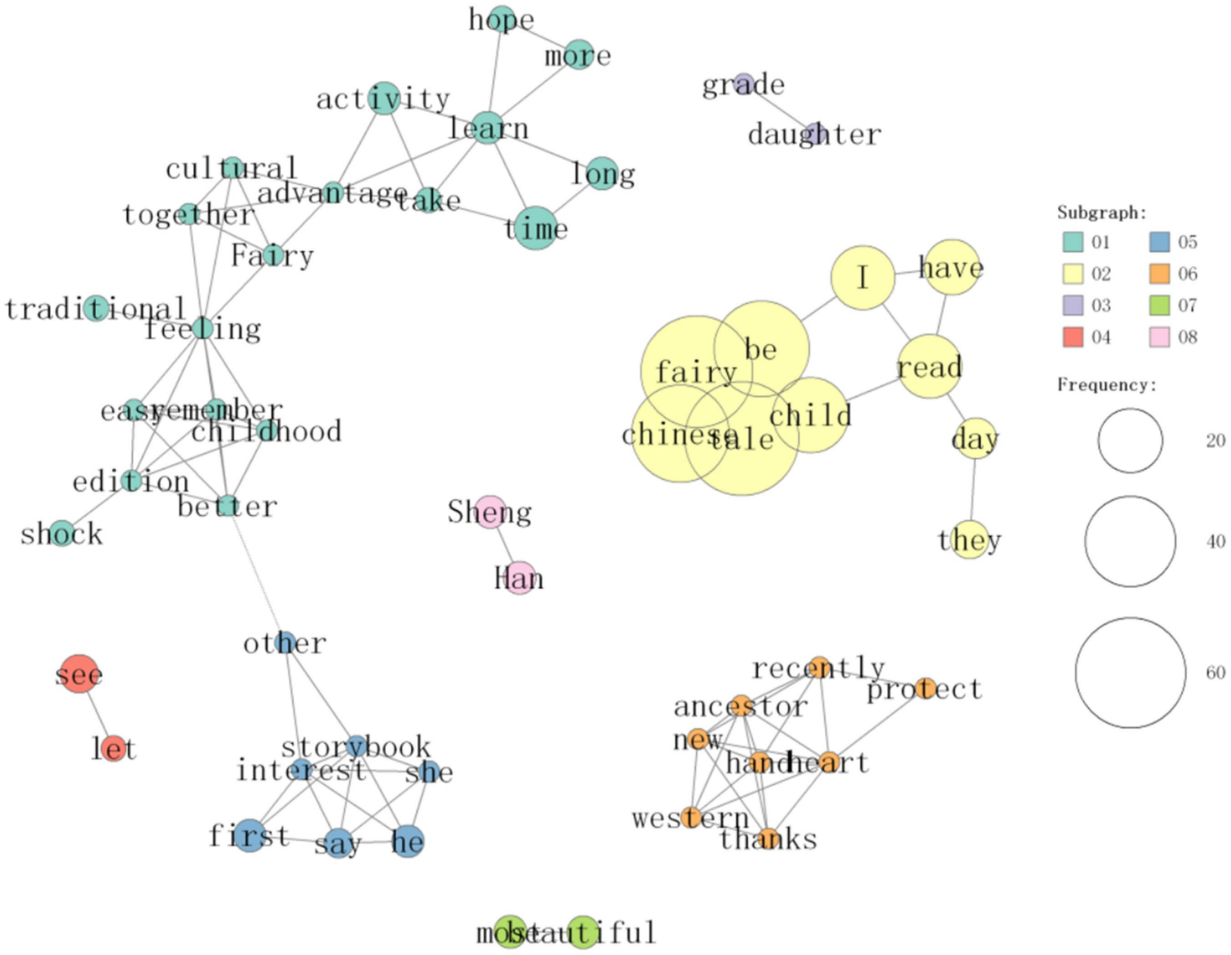
Display cooccurrence diagram of one of the word clusters.

In the word co-occurrence network, we can also see a group of pictures that show numerous words with strong nostalgia discourse for China, tradition, ancestors, etc., as shown in Figure 5.

These words show that readers of fairy tales have strong nostalgia for their own country’s fairy tales, they try to learn about the fairy tales handed down by their ancestors, some people try to have aspirations by learning about their ancestors’ reading experiences in the past, and they are eager to develop their own country and nation’s experiences through personal development to express their yearning for a better future. This evidence suggests that the nostalgic drive within “psychological ownership” is a significant thematic component in adult purchasers’ motivations and their discursive construction of children’s reading experiences with Chinese fairy tales.

### Psychological ownership

Psychological ownership manifests the extension of self-identity through psychologically owning a collection of tangible and intangible targets as ‘mine’ (Lin et al., 2022). In this inquiry, psychological ownership refers to one’s possessive feeling that the site is ‘mine’ (Lin et al., 2022). These include having a place, self-efficacy, self-identity, responsibility, and territoriality (Lin et al., 2023). The theory posits three routes for developing a possessive feeling: exercise of control, intimate knowledge, and investment of the self (Pierce et al., 2003). Psychological ownership, as a subjective sense of possession, has been widely applied in consumer behavior research. Rogers (2021) points out that even in the absence of legal ownership, individuals may develop psychological ownership over rented or shared items through pathways such as control, familiarity, and self-investment, thereby influencing their attitudes and behaviors. Li et al. (2021) further validate the role of psychological ownership in customer behavior, finding that self-image congruity and functional congruity can enhance customers’ sense of psychological ownership toward a brand through “impression in memory.” These studies provide theoretical support for understanding how adults articulate a sense of “psychological ownership” toward texts through self-reported emotional engagement and memory construction, and how they discursively relate this to their co-reading intentions.

The five attributes of psychological ownership include possession of a location, self-efficacy, self-identity, responsibility, and territoriality (Lin et al., 2023). Next, we explore the influence of fairy tale reading from the perspective of psychological ownership, with a focus on how these elements shape parents’ purchasing behaviors and co-reading practices. To ensure systematic analysis, we operationalized these five constructs using a dictionary-based quantitative content analysis. We developed specialized coding dictionaries (e.g., “childhood,” “memories,” “grandfather” for nostalgia; “inheritance,” “nurture” for responsibility), enabling a formal quantification of these dimensions across the 1,428 reviews. Unfolding these motivational factors lays the groundwork for understanding the psychological possession of fairy tales.

(1) Connections to the past. Intimate knowing pushes individuals to “come to find themselves psychologically tied to things because of their active participation or association with those things” (Pierce et al., 2003). To validate the identification of this construct, we conducted an inter-rater reliability check. Two independent researchers coded a randomized subsample of 300 reviews based on predefined operational definitions. The analysis yielded a high percentage agreement of 96.33% and a Cohen’s Kappa of 0.458. According to the benchmarks established by Landis and Koch (1977), a Kappa value of 0.458 indicates moderate agreement. It should be noted that the Kappa value is mathematically suppressed by the extreme scarcity of the target construct in the sample (nostalgic reviews accounting for only 3–4%), a recognized statistical phenomenon known as the “prevalence problem” or “Kappa paradox” (Cicchetti and Feinstein, 1990). Given this context, the high percentage agreement, complemented by a systematic review of the discordant samples, suggests that our coding framework serves as a preliminary basis for exploratory thematic mapping. Further examination of the 11 discordant samples revealed that discrepancies mainly centered on the criteria for “time dimension” and “emotional intensity”: Coder B was more inclined to classify long-term waiting (e.g., “watched for years,” “in cart for a year”) and emotional resonance (e.g., “moved to tears”) as nostalgic expressions, while Coder A required more explicit childhood memories or past-present contrasts. These discordant samples were independently arbitrated by a third researcher. Through triadic discussion, a final consensus was reached and applied to the subsequent analysis. Our full-corpus analysis identified “Connections to the Past” as the most prominent dimension, occurring 113 times.

The findings underscore that the discourse of adult purchasers frequently highlights an active interest in the culture and history behind these tales. “I find my childhood in it, all the folk stories my grandfather told me when I was a son’s age.” (R11) “I always miss the 365 nights when I was a child, and this big book just makes up for the regrets of my childhood. Wait for the baby to grow up, estimated at least five years old, to start the wonderful time of one story a day!” (R101) “Reading such stories makes me miss the image of my grandfather telling me stories when I was young, which is very warm. This should be the happiness that books can bring us.” (R325).

(2) Self-efficacy. Self-efficacy concerns one’s belief in ability to successfully executing actions to perform a task effectively (Avey et al., 2009). It reflects what it feels like to be in control “to meet my needs; being the cause of one’s control or actions results in feelings of efficacy and pleasure and creates extrinsic satisfaction as certain desirable outcomes are acquired. The desire to experience causal efficacy in altering the environment leads to attempts to take possession and to the emergence of feelings of ownership (Pierce et al., 2003). Our quantified textual analysis revealed 14 narratives consistent with a sense of self-efficacy in facilitating the co-reading process. For example, reviewers frequently express enjoyment in structuring the reading routine, such as reading one story a day. “My 11-year-old son could not sleep or eat enough to read it to me. He could not help but read it to me with emotion! He had never shown that he truly liked the books before. I read the recommended article, very moved, worthy of the craftsman!” (R22) “I would like to waste this wonderful weekend on reading, on parent–child time.” (R625).
(3) Self-identification Avey et al. (2009) remarked, individuals often rely on their interactions with tangible and intangible possessed targets to reshape their personal identities. Self-identity provides individuals with a source to “know themselves, define themselves, and express their self-identity to others” (Li et al., 2021). In our dataset, self-identification keywords (e.g., “national characteristics,” “own culture”) appeared 66 times. For adult readers, the language used in reviews suggests that they frame these books as cultural symbols that reaffirm their identities. “This book brings us warmth and strength.” (R28) “The pictures inside also have memories of my childhood.” (R63) “I will take her with me to read our own fairy tales every day, and may she be nourished by good myths!” (R15).
(4) Responsibility experienced responsibility and stewardship. Psychological ownership for a particular target may also promote feelings of responsibility that include feelings of being protective, caring, and nurturing and the proactive assumption of responsibility for that target (Pierce et al., 2003). This dimension was identified 20 times in the reviewers’ discourse, reflecting a felt duty to transmit culture to the next generation. “Growing up with the baby is the best company, and the baby will have something to talk about” (R1230).
(5) Sense of territory. In the territorial imperative, Ardrey attempts to demonstrate that “man is as much a territorial animal…” Home ownership has likewise been argued to satisfy the human need for having a place—my place (Tuttle, 1968). Personification of owned objects serves to promote security, identity, and individualism, each of which is important because it represents freedom of self-determination (Pierce et al., 2003). Our findings identified 4 explicit mentions of territoriality/defensiveness, which, though less frequent, carry significant emotional weight. Our findings suggest that adult purchasers frequently express a desire to associate their reading with the nation, framing fairy tales as a way of extending identity in their public reviews. In the review of Chinese Fairy Tales, taking the topic of “fairy tales” as an example, the author uses KH-Coder’s “KWIC Concordance” function to extract the word “Chinese” from 32,201 characters in 1,428 reviews. The number of words shared by fairy tales in the review was 184. Among them, the number of cooccurrences with “fairy” reaches 66, as shown in Table 2: KH-Coder has recently been shown to quickly code, network and cluster large textual corpora health-equity concepts embedded in physical-medical integration policies have been uncovered (Zhao et al., 2024); the construction of national identity in “dual-carbon” news has been traced via co-occurrence networks (Li and Zhao, 2025); and mental-health narratives have been illuminated through clustering techniques (Muramatsu, 2024). These precedents confirm the tool’s reliability across macro-policy and micro-emotion analyses and thus furnish methodological support for the present fairy-tale review investigation. In KH co-occurrence statistics, the score usually refers to the “Mutual Information” score, which is a statistic that measures the strength of the co-occurrence relationship between two words. A mutual information score can reflect the difference between how often two words occur together and the product of how often they occur independently. Specifically, if two words occur more often together than they do independently, then the mutual information score between the two words will be higher, indicating a strong correlation between them. The MI score values of these words were calculated together with “fairy tale.” The position relationship between “China,” “classic” and most co-occurrence words and keywords is relatively fixed, whereas words such as “story” and “like” generally appear after the word. In the specific comments, we can see several comments linking fairy tales with the motherland: “In this set of books, we can see our unique folk customs and art materials of the past dynasties, which will be beautiful for children’s memory.” (R17) At the same time, they also make a common criticism of fairy tales that are not their own country and their own people; for example, “It hurts to see children’s books westernized on the shelves at home.” (R17) “At a time when Western fairy tales such as Snow White are rampant, such books are badly needed, thanks to Han Sheng editors’ efforts” (R16). As Chinese people, we should not just try to steal foreign fairy tales. China’s own fairy tales are what we need to understand most.” (R28).

**Table 2:** The distribution of high-intensity co-occurrence words in fairy tales.

No	Word	POS	Total	LT	RT	L5	L4	L3	L2	L1	R1	R2	R3	R4	R5	The score
1	Fairy	Noun	66	4	62	2	0	2	0	0	59	2	0	1	0	61.317
2	Tale	Noun	73	5	68	1	2	0	2	0	0	64	2	0	2	34.767
3	Traditional	Adj	35	22	13	3	2	0	0	17	11	1	0	0	1	29.800
4	Culture	Noun	37	3	34	0	0	2	1	0	21	12	0	1	0	28.417
5	Story	Noun	28	5	23	1	1	2	1	0	16	2	1	3	1	19.900
6	Child	Noun	36	16	20	7	3	5	1	0	7	1	3	3	6	14.767
7	Very	Adv	31	21	10	4	1	9	4	3	0	3	1	4	2	12.283
8	Book	Noun	27	14	13	4	1	3	6	0	0	0	2	6	5	8.217
9	Good	Adj	22	14	8	6	2	2	1	3	0	0	2	1	5	7.783
10	Style	Noun	8	0	8	0	0	0	0	0	7	1	0	0	0	7.500

Linking fairy tales with national and national emotions mainly stems from the fact that “psychological ownership can also produce a sense of territory” (Lin et al., 2023). Research reveals that psychological ownership is associated with a sense of connection with one’s country through the reading of native fairy tales, a sentiment that can develop into a sense of territoriality. Territoriality essentially differs from having a place, although the latter is promotion-driven (Avey et al., 2009). In contrast, territoriality is prevention oriented, focusing on ‘the use of an external reference for territoriality and defensiveness’ (Avey et al., 2009). In this vein, feelings of territoriality compel individuals to fear that the owned target may be affected by external entities, thereby eliciting defensive endeavors (Avey et al., 2009). The study revealed that fairy tale readers, especially those who strongly believe that the book embodies the characteristics of the country’s own people, made efforts to protect their native fairy tales. They indicated their efforts in promoting the reading of these fairy tales, including the purchase, recommendation and reading of these fairy tales, actively protecting the purity of their country’s fairy tales and restoring cultural identity. For some readers, they object to forgetting any historical stories, including traditional festivals. “Chinese people cannot forget tradition, the traditional stories have great significance, even myths and legends, also has far-reaching significance; children’s hearts can not only Christmas, Avalokitesvara Bodhisattva, jade Emperor, too white Venus, the land of the father-in-law these should go deeper into the child’s heart” (R107). “Highly recommended, especially good book, the quality of the book, the arrangement, the composition and color of the color picture, all aspects reflect the classical Chinese beauty and strong national characteristics, worth reading and collecting” (R156).

Publishers could do a better job embedding nostalgic appeals in their fairy book offerings to evoke nostalgia. This method can cater to adult purchasers’ nostalgic emotions in promoting the consumption of books, which may theoretically contribute to the construction of a more supportive home environment for children’s reading—a speculative hypothesis that warrants future empirical verification, though actual impacts on children’s behavior require further empirical observation as our current data reflects parental discursive intentions rather than direct behavioral effects. Similarly, in the design of fairy tale books, readers should be given a control lever, whether in the purchase process or the reading process, so that readers can better reflect their sense of participation in reading. Designing some evaluation levels after reading fairy tales is a way to meet this increasing need for control. More importantly, since fairy tales are about telling Chinese stories, in China, telling Chinese stories well has become a central concern of local authorities. Thus, cultivating a sense of “home/hometown” through fairy tales books is closely related to taking a place in developing psychological ownership. Especially in Chinese culture and traditions, homes/hometowns not only represent places for dwelling and gathering but also symbolize belonging, identity, and the inner self’ (Yin et al., 2022).

Furthermore, these findings carry significant practical implications. From the perspective of educational psychology, our findings provide a mapping of parents’ nostalgic motivations which can offer insights for educators. These elements might be leveraged to encourage intergenerational dialogue, potentially supporting the transition from passive reading to active cultural transmission, though the efficacy of such interventions requires further empirical testing. In the domain of cultural psychology, stakeholders can explore these psychological ownership mechanisms as potential discursive tools to encourage children’s cultural identity; however, the actual impact on children’s long-term reading habits remains a hypothesis for future research.

It is crucial to clarify the analytical scope of this study. The present methodology relies on a dictionary-based quantitative identification (validated by manual reliability checks) and the descriptive analysis of co-occurrence networks. Consequently, our findings map the discursive representation of nostalgia and psychological ownership within adult purchasers’ textual feedback. The analysis does not employ an inferential model to test causal relationships between these themes and behavioral outcomes, nor does it provide a psychometric validation of the psychological mechanisms involved. Therefore, all subsequent discussions regarding psychological mechanisms should be interpreted as textual patterns and self-reported tendencies rather than proven behavioral predictors.

### Limitations and future directions

Several methodological limitations of this study must be acknowledged. First, our methodological approach is primarily an exploratory mapping of discourse based on text mining, rather than a confirmatory test of psychological mechanisms. Because we did not employ structural equation modeling (SEM) or mediation analyses with quantified construct scores for each review, causal inferences regarding how psychological ownership “predicts” or “impacts” emotion cannot be definitively drawn. The constructs of nostalgia and psychological ownership were interpreted heuristically from textual expressions rather than measured directly via validated psychometric scales.

Second, our dataset relies on self-selected e-commerce reviews from Dangdang, which introduces inherent sampling biases. E-commerce reviews are frequently influenced by external confounding factors, including promotional campaigns (e.g., discounts), expectations of platform rewards, and social desirability biases. Consequently, the expressed sentiments might overrepresent highly satisfied or highly dissatisfied purchasers, limiting the broader generalizability of the findings.

Future research should address these gaps by moving beyond textual exploration to empirical validation. We recommend integrating the five attributes of psychological ownership into a formalized measurement model and testing it via quantitative surveys or controlled behavioral experiments to systematically isolate genuine psychological ownership from general cultural nostalgia.

## Data Availability

The data set is available via restricted access at: https://zenodo.org/records/17149179?token=eyJhbGciOiJIUzUxMiJ9.eyJpZCI6ImJhNjkyNTMzLTc1ZWUtNGYzMC1hOGNlLTNkOTdjNTVjMWM0MCIsImRhdGEiOnt9LCJyYW5kb20iOiI5NDg5ZWViNjEwYzhhNzdhNzAxZDZkOTcyYTRjMDJmMCJ9.0rwK6T0f6M6qVY73ABQcwXancwVt4rZ8IVZhcfHQFqE5utsose6dG-hpRBcFamBGF1-dWoAdCkJ5VkR2TL7pSg.
